# The impact of patient safety culture on handover in rural health facilities

**DOI:** 10.1186/s12913-018-3708-3

**Published:** 2018-11-26

**Authors:** Donella Piper, Jackie Lea, Cindy Woods, Vicki Parker

**Affiliations:** 1 UNE Business School University of New England, Armidale, NSW 2351 Australia; 2 School of Health, University of New England, Armidale, NSW 2351 Australia

**Keywords:** Patient safety, Handover communication, Rural health services, Patient safety culture

## Abstract

**Background:**

Effective handover is crucial for patient safety. Rural health care organisations have particular challenges in relation to handover of information, placing them at higher risk of adverse events. Few studies have examined the relationship between handover and patient safety in rural contexts, particularly in Australia. This study aimed to explore the effect of handover on overall perceptions of patient safety and the effect of other patient safety dimensions on handover in a rural Australian setting.

**Methods:**

A cross-sectional online survey using The Agency for Healthcare Research and Quality Hospital Survey on Patient Safety Culture was implemented across six rural Local Health Districts in NSW, Australia and resulted in 1587 respondents. Hierarchical multiple linear regression analysis was conducted to account for the nested nature of the data. Models were developed to assess the effect of handover on patient safety perceptions, and the effect of other patient safety culture composites on handover variables. Open-ended questions about patient safety were inductively analyzed for themes. Quotes from the handover theme are presented.

**Results:**

All models were significant overall (*p* < .001), with explanatory powers ranging from 29 to 48%. Within rural health settings, effective handover is significantly related to patient safety perceptions (*R*^*2*^ = .29). A strong teamwork culture and management support culture was found to enhance effective handover of patient information (*R*^*2*^ = .47), and effective handover of personal responsibility (*R*^*2*^ = .37). A strong teamwork, management support, and open communication culture enhances handover of department accountability (*R*^*2*^ = .41).

Despite the implementation of standardised communication tools and frameworks for handover, patient safety is compromised by inadequate coordination, poor or absent documentation between departments, between other health care agencies and in transfer of care from acute facilities to primary/community care.

**Conclusion:**

Approaches to handover need to consider the particular challenges associated with rurality and strengthening elements found to be associated with increased safety, such as a strong teamwork and management culture and good reporting practices. Research is required to examine how communication at transition of care, particularly between facilities, is conducted and ways in which to enhance patients’ and families’ participation.

**Electronic supplementary material:**

The online version of this article (10.1186/s12913-018-3708-3) contains supplementary material, which is available to authorized users.

## Background

The term clinical handover is used in Australia to refer to ‘the transfer of professional responsibility and accountability for some or all aspects of care for a patient, or group of patients, to another person or professional group on a temporary or permanent basis’ [1]. The link between ineffective handover and patient safety in Australia has been previously documented [2–4]. Summarizing recent findings, Manias and colleagues [5 at 81] highlight the fact that ineffective communication during handover ‘is the most common cause of catastrophic or sentinel events in hospitals’, leading to communication at handover being identified as a key safety and quality issue currently being addressed by health service regulators and providers [5]. Effective handover is therefore essential to the quality and safety of care.

In terms of the impact, if any, of *rurality* on handover in Australia, there is evidence to suggest that the risks associated with handover communication are higher in rural areas because of additional challenges faced by rural facilities [2]. The vast geographical distances within Australia has resulted in some states having a *hub and spoke model* for delivery of care, where patients from rural areas are frequently transferred to and from larger facilities based in larger metropolitan and/ or regional centres for treatment. There is a need for staff to communicate and handover care across facilities by telephone or other IT means, which provides for high risk in communication of care-related information [2]. As well, differences in the staffing and skill mix within rural facilities plus a lack of resident medical staff, and problems with recruitment and retention of experienced staff when compared with metropolitan health services, compound the risks associated with quality and safety in rural health environments. Not surprisingly, given these additional challenges, Australian research focused in rural areas identifies handover as an issue in need of improvement [6].

Despite these additional challenges, most handover studies conducted with at least some rural Australian participants don’t analyze findings based on rurality, or if they do, they only study a small cohort of specific types of patients in a small number of settings, and focus on different types of handover. In addition, they all concentrate on the communication and information aspects of various types of handover and not the transfer of accountability and responsibility aspects, making it difficult to make generalizations about handover and its relationship with patient safety in rural Australia.

For example, Manias et al. [5] surveyed a variety of Australian health professionals from different disciplines and geographical locations, including rural health professionals, about their experiences of all types of handover including for example: shift-to-shift handover, nursing to medical handover, and inter and intra facility handover. Participants cited the following adverse events related to poor handover: ‘delays in treatment or procedure, or, prolonged treatment or procedure; lack of monitoring information given on clinical assessment, leading to patient deterioration; errors involving medications; patient falls; disruptive, aggressive behaviour and confused state leading to injury; putting patients at risk of infection and putting infants at risk’ [5]. Furthermore, participants were able to offer suggestions for improvements including using written documentation to complement oral communication, the use of checklist tools, education and mentoring by senior staff. Whilst there were significant differences between professions about their perceptions of the effectiveness of various types and aspects of clinical handover, data was not analyzed based on rurality, as the focus of this exploratory research was clinical discipline rather than setting.

Other researchers have reported on the adoption and/or implementation of standardized communication tools aimed at improving communication and thereby reducing the risk of adverse patient outcomes in rural settings, during specific types of handover; namely shift-to-shift and nurse-to-doctor handover in a large Victorian regional hospital [2], and for handover of acutely ill deteriorating patients transferring from a rural facility to a tertiary hospital throughout Western Australian country health service facilities [7]. Although the effect on patient outcomes was not assessed [2, 7], in the Victorian study nurses reported improvements in shift-to-shift and nurse-to-doctor handover after implementation of the standardized tool *and* communication training [2]. In the Western Australian study, there was early adoption of the tools and staff experiences of using the tools were reported as positive [7].

Other rural Australian handover studies focus on the effect on communication and information of different methods for conducting handover. For example, Bradley and Mott [8] evaluated the introduction of nurse-to-nurse bedside handover within the acute ward in three hospitals in rural South Australia. The authors found that both patients’ and nursing staff satisfaction increased with bedside handover compared to the previously implemented closed-door handover process. Manias et al. [9] examined clinical handover conducted by telephone for inter-facility transfers of patients transferred from rural to a metropolitan hospital via air transport. Overall findings suggested: ‘Crucial information was missing from calls, which may have contributed to delay and inappropriate delivery of care’ [9 at 373]. A point of difference between rural and urban facilities was identified by Fassett et al. [10] who found the prevalence of handover between after-hours and day personnel or medical morning handover, to be less likely in rural hospitals, in the hospitals surveyed.

Much of the rural handover literature concentrates on the communication aspects of inter-facility handover for specific groups of patients. In Australia, Drabsch et al. [11] report on the implementation of a hub (regional)-and-spoke (rural) multidisciplinary team model of care for orthogeriatric in-patients, who had fractured a lower limb from a fall, admitted to facilities in the Western New South Wales Local Health District. In terms of handover, the authors found a senior multidisciplinary team working between regional and rural hospitals providing inter-professional and collaborative care increased adherence to clinical practice guidelines. In turn, this improved handover from regional to rural facilities, with multidisciplinary handover promoting communication between care providers across the care continuum; leading the authors to conclude the model could improve clinical care for other frail older inpatients transitioned from regional to rural facilities.

In America, Agvtis et al. [12] and Kappel et al. [13] both tested the effects of a combination of a Rural Trauma Team Development Course (RTTDC) and communication training on the inter-facility transfer times of rural trauma patients. Both studies reported that personnel trained in the RTTDC course only, as well as those completing the course with an additional communication module, reported shorter times for a decision to transfer the patient to a larger hospital, with those receiving both components having the lowest times in relation to the decision to transfer (although this was not significant).

Johnson et al. [14] examined information exchange in handover between physiotherapists, between physiotherapists and other health care professionals, and between physiotherapists and family members, in the care of elderly (over 65) patients with hip fractures, who had initially been admitted to two Canadian rural hospitals, during all points in the care continuum. Findings revealed that handover was less successful when access, retrieval, transfer, flow and exchange of information were untimely or incomplete. The researchers found that ‘inadequate handover compromised continuity of care, delayed progress in rehabilitation, and resulted in families’ missing information of vital importance to their caregiving role’, leading them to conclude ‘that a multi-directional exchange of information was needed between patients, families and health care providers across all care settings’ [14 at 266]. In terms of improving more complete information, Murad et al. [15] examined the implementation of a mobile web-based software to share patient information between emergency service personnel and hospital emergency staff in rural Minnesota. Whilst further research was needed, initial findings suggest the software can help ensure more complete information during handover from emergency service personnel to emergency staff in hospitals and worked best for patient pre-registrations and early notifications, especially where there were long transport times and more severe incidents.

In addition to this body of literature on information and communication aspects of rural handover, there exists a small body of international literature reporting on the handover dimension of the Agency for Healthcare Research and Quality (AHRQ) Hospital Survey on Patient Safety Culture (HSOPSC) in rural settings. The HSOPSC contains 42 items to measure staff opinion on 12 patient safety culture dimensions, including *handover* (the other 11 dimensions are: teamwork culture within and across units (× 2), management expectations culture, organisational learning culture, management support culture, patient safety culture, communication culture (× 2), reporting culture, staffing culture and non-punitive response to errors [16]).

We identified nine papers reporting on the implementation of the survey across a range of rural settings in various geographical locations including, Portugal (Fernendez et al. [17]), Slovakia (Mikusova et al. [18]), and the United States (Lee et al. [19], Adams-Pizarro et al. [20], Hannah et al. [21], Jones et al. [22], Klingner et al. [23], Pagan-Sutton et al. [24], and Tupper et al. [25]).

In Europe, Fernandes et al. [17] report on the implementation of the survey in a rural setting in Portugal. However, the article is not available in English. Mikušová et al. [18] implemented the HSOPSC in 3 hospitals from the Trnava region of Slovakia. In addition to providing a baseline measure of patient safety culture, the analysis revealed that nurses’ and doctors’ opinion on seven of the 12 dimensions of patient safety culture differed. The areas that differed significantly were communication culture, reporting culture, handover culture, patient safety culture and management support culture. Doctors’ opinions were significantly more positive in these dimensions compared with nurses.

In the USA, in terms of the results from the implementation of the HSOPSC overall, papers report positive outcomes, across several of the survey’s dimensions, upon redeployment of the survey following a patient safety intervention. Summarising the studies implementing the HSOPSC in the USA, Croll et al. ([26] at 1) report that the HSOPSC has ‘been effective for planning, implementing, and evaluating targeted patient safety interventions in Critical Access Hospitals.’ Of note and applicable to the current study, are the conclusions drawn by the authors of the US studies in relation to clinical handover. Few of the studies elaborate beyond reporting the ranking of scores of handover at baseline and following implementation of an initiative to improve patient safety culture, none of which focus on handover itself. Studies identifying the ranking of the handover dimension consistently report handover to be one of the most negatively rated dimensions of patient safety culture [20–23, 25].

In contrast to previous research, Lee et al. [19] examined handover in terms of not only communication and information provision but also the aspects of transfer of responsibility and accountability, and analysed how the different dimensions of the HSOPSC are associated with clinical handover. They conclude that all aspects of handover are associated with perceptions of patient safety. Lee et al. [19] also reported that a strong communication culture only partially improves handover of patient information, while a strong teamwork culture within departments and a strong reporting culture improve handover of personal responsibility. A strong teamwork culture across departments improved handover of department accountability whereas management support had a negative influence on handover of department accountability. However, Lee and colleagues did not conduct comparisons between rural and urban settings.

Overall, the literature on handover in rural settings is sparse and often limited to specific patient populations, clinical specialties, and types of handover as well as being skewed to the information and communication aspects of handover. No studies have explored the relationship between patient safety perceptions and patient safety culture on effective handover of information, responsibility and accountability, in a rural Australian setting. This study aimed to explore the effect of handover on overall perceptions of patient safety. However, handover is also a vital part of patient safety culture (PSC) (one of 12 PSC composites in the HSOPSC). Thus, this study also aimed to explore the effect of other PSC composites on handover in a rural setting.

## Methods

### Study design

A cross-sectional online survey using the AHRQ HSOPSC was conducted during March–April of 2015. The advantage of a cross-sectional study is data is collected at a specific point in time and provides a snapshot in time to examine the relationship between variables of interest. However, cross-sectional studies cannot determine cause and effect [27].

### Sample and setting

Public health services in New South Wales (NSW) are divided into 15 Local Health Districts (LHDs) or networks. Eight districts cover the greater Sydney metropolitan region, and seven cover rural and regional NSW [28]. Six of the seven rural and regional LHDs in NSW agreed to participate in the study. Participating LHDs ranged in size from seven to 40 facilities. Participants were staff employed in public health services across rural LHDs of NSW, Australia, including health professionals, managers, administrative support staff, Aboriginal liaison officers and others. Inclusion criteria were staff who had worked within the District for at least 6 months, in any capacity, whether clinical or non-clinical. Response rates from the six LHDs ranged from 2 to 28.5%. This sample represents between 4 and 5% of the LHDs’ staff population. Overall, 1663 participants submitted a questionnaire, 5% (*N* = 76) were excluded due to excessive missing data (> 50%), leaving 1587 responses.

### Data collection tool

The HSOPSC contains 42 items to measure staff opinion on the following 12 dimensions of patient safety culture: teamwork within units, supervisor/manager expectations and actions promoting patient safety, organisational learning—continuous improvement, management support for patient safety, overall perceptions of patient safety, feedback and communication about error, communication openness, frequency of events reported, teamwork across units, staffing, handover and transitions, and, non-punitive response to errors [29]. Two composite items that were not deemed necessary for this study, organisational learning—continuous improvement and staffing, were removed from the questionnaire as per user guide instructions [30]. The HSOPSC also asks respondents to provide an overall safety rating to their department and to identify the number of adverse events reported by them in the last 12 months. All HSOPSC item responses are scored on a five-point Likert type scales of agreement (strongly disagree to strongly agree) or frequency (never to always) [29].

The HSOPSC was modified to adjust the language of the questionnaire slightly to suit the Australian rural context. For example, Physician was replaced with Doctor, hospital was replaced with facility, unit was replaced with department, and handoffs was replaced with handover. For *Frequency of Events Reported*, the questions in the HSOPSC were replaced with the following questions:

1. How often is a near miss reported?
2. How often is a Severity Assessment Code (SAC) 4 reported?
3. How often is a SAC 3 reported?
4. How often is a SAC 2 reported?
5. How often is a SAC 1 reported?

A ‘near miss’ is categorised as ‘Any event that could have had adverse consequences but did not. An arrested or interrupted sequence where the incident was intercepted before causing harm e.g. an incorrect medication added to an infusion but not administered’ [31]. Severity Assessment Codes (SAC) are used to rate the severity of a patient safety incident, with SAC 1 denoting the most serious clinical incident/near miss that could have caused serious harm or death, SAC 2 denotes moderate harm, and SAC 3, minimal harm [31]. The demographic questions were tailored to suit the rural NSW system of health service delivery.

A final free text comments section was provided for participants to write any comments about patient safety, error, or event reporting in their facility.

### Data collection

The online survey, hosted by Survey Monkey, was available to staff in the six participating LHDs. Associate investigators were engaged at each of the sites to facilitate implementation. Associate investigators were executive directors of the LHDs, who were mostly directors of clinical governance, and in some sites cluster or area managers were also involved. The Associate investigators sent an invitation via email inviting eligible staff to participate. Included in the invitation was the link to the online anonymous survey. A reminder was emailed to potential participants once over the period in which the survey was opened. Associate investigators did not have access to survey responses.

### Ethical issues

The first page of the online survey contained a participant information sheet that explained the study and invited voluntary participation. Potential participants were given the option to consent to complete the questionnaire and by clicking the consent button they were taken to the questionnaire - or not consent, whereupon the questionnaire closed and they were thanked for their time. All questionnaires were anonymous, with no personally identifying information collected. The study received ethics approval from the Hunter New England Human Research Ethics Committee. In addition, site-specific approval was sought and granted from all participating LHDs.

### Measures

#### Covariates

Two facility characteristics relating to facility type and LHD were included as baseline covariates as these factors were expected to affect perceptions of patient safety culture. For example, large regional principal and tertiary referral or specialist hospitals may experience more adverse patient safety incidents because they serve a larger, more diverse and complex patient population than smaller rural facilities. The frequency distribution of covariates is reported in Table 1.

**Table 1:** Frequency distribution of covariates

Health service characteristics	N	%
Facility Type^a^		
Multi-purpose Service	178	11.7
Community Health	362	23.8
District Hospital	522	34.4
Rural Referral Hospital	239	15.7
Tertiary Referral Hospital	52	3.4
Other	165	10.9
Local Health District^b^		
A	301	19.8
B	263	17.3
C	304	20.0
D	171	11.3
E	73	4.8
F	407	26.8

^a^69 persons did not report Facility Type

^b^68 persons did not report Local Health District

### Patient safety perceptions

*Patient Safety Perceptions* consists of four items that measure participants’ agreement that ‘our procedures and systems are good at preventing errors from happening’, ‘it is just by chance that more serious mistakes don’t happen around here’ (reverse coded), ‘patient safety is never sacrificed to get more work done’, and ‘we have patient safety problems in this department’ (reverse coded).

### Handover

Following the method of Lee et al. [19], four survey items relating to handover and transitions of care were used in the analyses. *Handover of patient information* consists of two items, ‘problems often occur in clinical handovers across departments’ (reverse coded) and ‘important patient care information is often lost during clinical handover’ (reverse coded). *Handover of personal responsibility in shift changes* is measured by the item, ‘clinical handover is problematic for patients in this facility’ (reverse coded). *Handover of unit accountability* is measured by the item, ‘things fall between the cracks’ during clinical handover from one unit to another’ (reverse coded).

### Patient safety culture

Two composite scales measure Communication culture, *communication openness* and *feedback and communication about error*. Two composite scales measure Teamwork culture, *teamwork within units* and *teamwork across units*. Reporting culture is measured by the composite *frequency of events reported*. Three composites measure Supportive management action, *management support for patient safety*, *supervisor/manager expectations and actions promoting patient safety*, and *non-punitive response to error*. The items in the HSOPSC survey that represent each of these composites are reported in Table 2.

**Table 2:** HSOPSC survey items for each patient safety culture composite

Communication Openness	
1. Staff will freely speak up if they see something that may negatively affect patient care.	
2. Staff feel free to question the decisions or actions of those with more authority.	
3. Staff are afraid to ask questions when something does not seem right. (reverse coded)	
Feedback & Communication About Error	
1. We are given feedback about changes put into place based on incident reports.	
2. We are informed about incidents that happen in this department.	
3. In this department, we discuss ways to prevent incidents from happening again.	
Teamwork Within Units	
1. People support one another in this department.	
2. When a lot of work needs to be done quickly, we work together as a team to get the work done.	
3. In this department, people treat each other with respect.	
Frequency of Events Reported	
1. How often is a near miss reported?	
2. How often is a Severity Assessment Code (SAC) 4 reported?	
3. How often is a SAC 3 reported?	
4. How often is a SAC 2 reported?	
5. How often is a SAC 1 reported?	
Teamwork Across Units	
1. There is good cooperation among facility units that need to work together.	
2. Facility departments work well together to provide the best care for patients.	
3. Facility units do not coordinate well with each other. (reverse coded)	
4. It is often unpleasant to work with staff from other departments within the facility. (reverse coded)	
Management Support for Patient Safety	
1. Facility management provides an environment that promotes patient safety.	
2. The actions of facility management show that patient safety is a top priority.	
3. Management seems interested in patient safety only after an incident happens. (reverse coded)	
Supervisor/Manager Expectations & Actions Promoting Patient Safety	
1. My supervisor/manager acknowledges when he/she sees a job done according to established patient safety procedures.	
2. My supervisor/manager seriously considers staff suggestions for improving patient safety.	
3. Whenever pressure builds up, my supervisor/manager wants us to work faster, even if it means taking shortcuts. (reverse coded)	
4. My supervisor/manager overlooks patient safety problems that happen over and over. (reverse coded)	
Non-punitive Response to Errors	
1. Staff feel like their mistakes are held against them. (reverse coded)	
2. When an event is reported, it feels like the person is being written up, not the problem. (reverse coded)	
3. Staff worry that mistakes they make are kept in their personnel file. (reverse coded)	

### Data analysis

Negatively worded HSOPSC items were reverse scored, then items for each composite were summed to create composite subscales. The internal consistency of the patient safety composite scales was measured using reliability analysis. All scales showed a relatively high level of internal consistency and were all in the acceptable range of > .70 (Table 3), with Cronbach’s alpha ranging from α = .75 (*Handover of patient information*) to α = .89 (*Frequency of events reported*).

**Table 3:** Descriptive statistics and reliability analyses of the items in each patient safety culture composite

Patient safety culture composite items	N	Mean	SD	Cronbach’s alpha
Patient Safety Perceptions	1229	3.32	.82	.78
Handover of Department Accountability	1113	3.12	1.01	–
Handover of Personal Responsibility	1088	2.77	.96	–
Handover of Patient Information	1091	3.12	.89	.75
Management Support for Patient Safety	1103	3.46	.83	.79
Supervisor/Manager Expectations & Actions Promoting Patient Safety	1225	2.85	.43	.82
Non-punitive Response to Error	1227	3.15	.92	.82
Communication Openness	1176	3.49	.78	.77
Feedback & Communication about Errors	1180	3.50	.91	.84
Frequency of Events Reported	1031	3.76	1.00	.89
Teamwork Within Units	1240	3.79	.65	.80
Teamwork Across Units	1083	3.41	.75	.82

Hierarchical multiple linear regression analysis was performed using SPSS v23. The use of this technique takes into account the nested nature of the data; individuals nested within health facilities, nested within local health districts. Standard multivariate models are not appropriate for analysis of hierarchical data due to violation of the assumption of independence [32]. Hierarchical multiple regression was selected in order to evaluate the contributions of predictor variables in a sequential way, so that the relative importance of variables can be judged by how much it adds to the prediction of a criterion, as a means of statistical control, and to isolate predictors which have a significant influence on patient safety perceptions [33]. This technique allowed us to enter the variables in a fixed order, controlling for the influence of the covariates and allowing us to isolate the effects of the predictors of patient safety perceptions. Preliminary analyses were conducted to ensure the assumptions of normality, linearity and homoscedasticity were not violated. Multicollinearity was checked among the covariates and predictors using the variance inflation factor (VIF). The VIF was below 3.0, denoting any significant relationships found are not inflated by correlations between the predictor variables [34]. Tolerance was above .10 indicating that multiple correlation with other variables is low.

To assess whether handover of patient information, personal responsibility and department accountability are associated with patient safety perceptions, the two facility covariates were first entered into the regression model as baseline predictors of patient safety perceptions. Then each handover variable was entered into the regression model.

To assess the effects of other patient safety culture composites on each handover variable, the two facility covariates were first entered into the regression model as baseline predictors of each handover variable, followed by the respective patient safety culture composites.

Qualitative free text data were analysed using qualitative content analysis. Comments were read several times to identify key thoughts and concepts in the data relating to handover and patient safety culture, and codes were applied. Codes were then grouped into higher-order categories to collapse data with similar meaning. The categories were then abstracted and described with a content-based code. The categories are described as a summary of the participants’ statements, and representative quotes were identified that best reflect the statements.

### Sample size analysis

Sample size analysis for multiple regression was conducted in G*Power to determine a sufficient sample size using an alpha of 0.05, a power of 0.80, and a small effect size (f^2^ = 0.02). Based on the aforementioned assumptions, the desired sample size was 904. This calculation was based on 11 independent variables and two control variables.

### Quantitative results

The characteristics of the respondents are set out in Table 4 below. Typically, the respondents: worked in district hospitals, community health facilities or multi-purpose services (MPS), psychiatric facilities, sub-acute, palliative, dialysis and other small facilities; had worked between 1 and 10 years at their current facility; worked only at one facility; worked as a nurse either across units within a facility or primarily in community, mental or allied health units. Nearly half of the respondents worked more than 40 h per week. The vast majority had direct interaction or contact with patients, were female and over one-third of respondents were aged 45–54, followed closely by the 55–64 age group.

**Table 4:** Demographic characteristics of respondents

Characteristics	%
No. of years spent at facility	
≤1	10
1–5	32
6–10	20
11–15	14
16–20	9
21+	15
Staff position	
Nursing	51
Allied health	20
Administration	9
Management	8
Other	7
Medical	4
Aboriginal Liaison Officer	1
Primary work area	
Many different units/no specific unit	19
Community health	17
Allied health	11
Mental health	11
General ward	7
Emergency	5
Obstetrics	5
Outpatients	5
Acute (surgical)	4
Acute (non-surgical)	3
Radiology	3
Sub-acute	2
Paediatrics	2
Intensive care	2
Rehabilitation	1
Pharmacy	1
Recovery	1
Pathology	1
Hours per week worked at main facility	
40+	49
20–39	45
≤20	6
Age of respondents	
65+	2
55–64	31
45–54	34
35–44	17
25–34	12
18–24	4
Gender	
Female	85
Male	15

The characteristics of those who responded to the initial survey invitation email were compared to those who responded to the later follow-up email reminder (see Additional file 1). The respondents did not differ in age, sex, type of facility worked in, hours worked per week, or main staff position. However, there were some differences in LHD, number of years worked in the facility, and primary work area.

All sub-scale responses were also compared between initial and later respondents, with only one difference noted. There was a small significant mean difference in the *Feedback and communication about error* sub-scale (mean difference 0.13), *p* = 0.017.

Table 5 reports the results of regression analysis of the relationship between patient safety perceptions and handover. Hierarchical multiple regression was used to assess the ability of handover measures to predict patient safety perceptions after controlling for LHD and facility type. LHD and facility type were entered at Step 1, explaining 9% of the variance in patient safety perceptions. After entry of the handover variables in Step 2, the total variance explained by the model as a whole is 28.5%, *F*(5, 1040) = 82.84, *p* < .001. The three handover measures explained an additional 28% of the variance in patient safety perceptions after controlling for facility type and LHD, *R* squared change = .28, *F* change (3, 1040) = 134.51, *p* < .001. In the final model, effective handover of patient information, personal responsibility and department accountability are all statistically significantly associated with patient safety perceptions, with *Handover of department accountability* recording a higher beta value (*beta* = .25, *p* < .001) than *Handover of patient information* (*beta* = .18, *p* < .001) and *Handover of personal responsibility* (*beta* = .16, *p* < .001), indicating a stronger association with perceptions of patient safety. These results strongly support that effective handover is related to patient safety perceptions.

**Table 5:** Hierarchical regression analyses on the impact of handover on patient safety perceptions

	Patient safety perceptions	95% CI
Control variables:		
Facility Type	−.03	−.05, .02
Local Health District	−.08^*^	−.05, −.01
Predictor variables:		
Handover of patient information	.18^***^	.09, .25
Handover of personal responsibility	.16^***^	.08, .20
Handover of department accountability	.25^***^	.14, .27
R^2^	29^***^	
R^2^ change	.28^***^	

Values in the table are standardized beta coefficients

*R*^2^ change = improvement in *R*^2^ when an additional predictor is added

Note: ^*^ *p* < .05, ^**^ *p* < .01, ^***^ *p* < .001

Table 6 reports the results of regression analysis of the relationship between patient safety dimensions and handover. Hierarchical multiple regression was used to assess the effect of other patient safety dimensions on handover after controlling for LHD and facility type. Each model follows the same Step 1 of entering facility type and local health district, and independent variables entered at step 2. In Model 1 LHD and facility type explained 1% of the variance in *Handover of patient information*. After entry of the patient safety variables, the total variance explained by the model as a whole is 47.7%, *F*(11, 884) = 73.25, *p* < .001. The patient safety variables explained an additional 47% of the variance in *Handover of patient information* after controlling for LHD and facility type, *R* squared change = .47, *F* change (9, 884) = 89.06, *p* < .001.In the final model, *Handover of patient information* is significantly positively associated with *Teamwork across departments* and *Management support for patient safety.* The data indicate that a strong teamwork culture and a strong management support culture enhances handover of patient information.

**Table 6:** Hierarchical regression analyses on the effect of other patient safety dimensions on handover

	Handover of patient information		Handover of personal responsibility		Handover of department accountability	
	Model 1		Model 2		Model 3	
	β	95% CI	β	95% CI	β	95% CI
Patient safety culture						
Communication openness	.02	−.07, .11	.04	−.05, .16	.10^*^	.03, .24
Feedback & communication on errors	.03	−.06, .11	.01	−.09, .11	−.01	−.10, .09
Teamwork within departments	−.07	−.23, .05	−.11	−.32, .11	.01	−.16, .17
Frequency of events reported	−.03	−.08, .02	−.04	−.09, .02	−.04	−.09, .02
Teamwork across departments	.55^***^	.57, .73	.45^***^	.09, .28	.46^***^	.53, 72
Management support for patient safety	.18^***^	.12, .28	.17^***^	.09, .28	.17^***^	.11, .29
Supervisor/Manager expectations & actions promoting patient safety	−.03	−.17, 07	−.03	−.21, .07	−.01	−.16, .13
Nonpunitive response to error	.04	−.03, .09	.05	−.02, .12	.01	−.06, .09
R^2^	.48^***^		.37^***^		.42^***^	
R^2^ change	.47^***^		.37^***^		.41^***^	

Values in the table are standardized beta coefficients

*R*^2^ change = improvement in *R*^2^ when an additional predictor is added

^**^ *p* < .01 ^***^ *p* < .001

In Model 2, LHD and facility type explained 1% of the variance on *Handover of personal responsibility*, while the model as a whole explained 36.8% of the variance, *F*(11, 879) = 46.61, *p* < .001. The patient safety variables explained an additional 37% of the variance in *Handover of personal responsibility* after controlling for LHD and facility type, *R* squared change = .37, *F* change (9, 879) = 56.86, *p* < .001. *Teamwork across departments* and *Management support for patient safety* have a significant positive association with perceptions of effective handover of personal responsibility, signifying that a strong teamwork culture and a strong management support culture enhances handover of personal responsibility.

In Model 3, LHD and facility type explained 1% of the variance on *Handover of department accountability,* while the model as a whole explained 41.5% of the variance, *F*(11, 888) = 57.19, *p* < .001. The patient safety variables explained an additional 41% of the variance in *Handover of department accountability* after controlling for LHD and facility type, *R* squared change = .41, *F* change (9, 888) = 69.47, *p* < .001. *Communication openness, Teamwork across departments* and *Management support for patient safety* are all statistically significantly associated with *Handover of department accountability*, denoting that a strong communication culture, teamwork culture and management support culture enhances handover of department accountability. In all three models, the higher beta values of *Teamwork across departments* supports that effective handover is more strongly related to a strong teamwork culture than a management support culture or a communication culture.

### Qualitative results

We received a total of 297 free text responses to the final survey question ‘Please feel free to write any comments about patient safety, error, or incident reporting in your hospital.’ Two of the researchers (JL and DP) blind coded the responses into themes. These researchers then compared themes, and differences in interpretation were negotiated until agreement was reached. The major theme was inadequate staffing, however responses also related to: facilities and equipment, incident reporting and management, workplace culture, teamwork, rural isolation and handover. In relation to handover, 19 participants provided comments. Because of this small sample size we did not compare across LHDs or collate characteristics of respondents.

The 19 open-ended responses relating to handover indicated that respondents believed that *patient safety is often compromised by communication* and poor handover of information occurs at various stages of a patient’s journey through the health system. Respondents highlighted the challenges associated with complex conditions, multiple numbers of multidisciplinary care providers, lack of time and non-adherence to reporting guidelines. Rural facilities and transfer between facilities were seen as creating higher risk for rural patients.

Within the context of complex, multidisciplinary care, poor handover communication was attributed to poor understanding of the roles of other health professionals and limited acknowledgement by some health staff of the care that other departments had delivered or could deliver to patients. This potentially resulted in delays in care and treatments thereby compromising patient safety. Further, patients themselves and their families are often not provided with essential information that would allow them to feel secure and participate in care.

*The biggest issues… are clinical handover for patients who have complex degenerative conditions, the inpatient care plan and discharge planning is poor… especially when more than one specialist medical team is involved… poor understanding of the roles of allied health and therefore when most appropriate to refer.*

Another issue of particular concern was the ability to successfully provide communication for vulnerable groups where there is a high likelihood of miscommunication, for example culturally and linguistically diverse groups and those with severely impaired hearing*.*

*Medical interpreters are needed for - adequate history of patient, correct information, explanation of diagnosis, treatment and medications. Increasing numbers of patients that refuse interpreters because they think they understand and practitioners that do not take responsibility to be sure the client has the correct information.*

Supports, such as interpreters are less likely to be available to smaller facilities. Many problems are seen to be exacerbated by the pressure of not having enough time; time to listen, plan and connect with others who need information in order to ensure continuity of care for patients.

Many respondents were concerned with the quality of handover communication between facilities within health districts, perceiving poor communication and poor or absent documentation as the main challenges for patient safety.

*There are major problems with communication between facilities… and this impacts greatly on patient safety.*

When patients are being transferred from one hospital to another for higher level care or as part of a discharge plan, the handover communication between facilities in rural areas involved a higher risk.

*Clinical handover is where most of the safety issues happen. If you are rural this happens in both directions so twice the risk.*

In addition, respondents commented that in their experience problems also appear to relate to a lack of organization and coordination of patient inter hospital transfers, particularly the inadequate documentation and fragmentation of providers between facilities.

*More emphasis on Doctors and medical staff completing patient’s notes/documentation electronically would be a great improvement on patient safety considerations and easy transfer of clinical information.*

Respondents recommended a need for more collaboration and communication between facilities, and between primary and community care settings, regarding handover of client care. This issue was described as *poor communication between acute setting and primary health,* where referrals are not received from discharging units or where the handover for patients being transferred to other health care facilities (for example from a major tertiary or regional referral hospital to smaller rural facilities or vice versa) was perceived as inadequate.

*Multiple IT systems that do not communicate with each other make it difficult to communicate care, document care where it is accessible to everyone across the continuum and follow up incidents and near misses…challenging.*

Many respondents commented on staffing issues within the rural environment and believed that there was a direct relationship between under-reporting of incidents and staffing problems, to problems with poor handover communication as the below quote illustrates.

*Incidents tend to be under reported… many staff are unaware of what type of incident needs to be reported… a small facility, we are typically understaffed… this exacerbates existing problems with providing clinical handover.*

Structured clinical handover practices within clinical units has reportedly improved patient safety and has assisted patients to be involved in their care*.*

*Bedside handover has been the best introduction to patient safety... This reduces medication error and ensures all treatments have been completed, or if not, allows the oncoming staff to pick up what has not been completed. The patient is also able to be involved in discussing their care at the bedside.*

However, one respondent commented that *established clinical handover procedures are almost never complied with*, and many other respondents believed that whilst good in theory, handover could be improved within and between interdepartmental facilities. That is handover for when care was being transferred to other departments or other health professionals on discharge from acute care areas or for procedures in other areas of the facility. As the following quote demonstrates, respondents acknowledged that this was an important point in the transition of care and improvement here would improve the communication with families regarding the transfer of care and avoid treatment delays for patients.

*A lot of work has been done around improving communication from inpatients to out, but patients continue to fall between the cracks.*

### Discussion

Through the use of the Agency for Healthcare Research and Quality (AHRQ) Hospital Survey on Patient Safety Culture (HSOPSC) this study explored the effect of handover on overall perceptions of patient safety, and the factors in healthcare organisations’ patient safety culture that may be associated with effective handover in the rural context. The results of this study show that effective handover of patient information, personal responsibility at shift change, and department accountability during patient transfers are significantly associated with patient safety perceptions. Within rural health settings strong teamwork across departments and management support culture were found to enhance effective handover of patient information and handover of personal responsibility. A strong communication culture, teamwork culture and management support culture enhances handover of department accountability.

A previous USA study on handover [19] using the HSOPSC found that communication openness was not associated with effective handover of patient information, however this was not tested with handover of department accountability. The current study found that communication openness was positively associated with effective handover of department accountability [19]. However, despite strategies put in place regarding systematic communication using standardised tools and frameworks for handover, respondents to this survey believed that patient safety is compromised by inadequate and disorganised coordination, and poor or absent documentation of handover communication particularly in the following transition points of care: between departments within the facility, between other health care agencies within the LHD as well as when care is transferred from acute facilities to primary/community care.

The current study shows a strong teamwork culture across departments enhances perceptions of effective handover of personal responsibility during shift changes. However, participants also noted that inadequate verbal handover communication and insufficient or missing documentation at these points in the transition of care poses risks to patient safety by causing delay in treatments, procedures and care by other health professionals and fails to involve the family in the transition of care process. A reporting culture was not associated with perceptions of effective handover of personal responsibility, whereas the opposite was found in Lee et al’s study [19]. Ineffective handover can create information gaps, errors in patient care and adverse events [35, 36]. Manias et al. [5] also found that inadequate handover communication and documentation puts patients at increased risk for adverse events because of delays in treatment or procedures. Further, Manias et al. [5], when examining clinical handover conducted by telephone for inter-hospital transfers of patients from rural to metropolitan hospitals, suggest that crucial information can be missing. The potential impacts of lack of crucial information during handover may be felt at a number of levels including inadequate and inappropriate care resulting in patient harm, and breach of safety and quality standards [5]. Manias and colleagues [5] made a number of recommendations for improvement around the use of structured checklists, compliance with standards and procedures and access to and clarity of information. Similar findings in an allied health (physiotherapy) setting [14], has led to the conclusion that information must be shared between all stakeholders across all care settings.

The results of the current study indicate that strong communication, teamwork across departments and management support for patient safety enhanced effective handover of department accountability. Whereas Lee et al’s [19] study found that management support was negatively associated with perceptions of effective handover of department accountability. It is possible that in smaller rural health facilities staff are more aware of and better able to observe management support during routine interactions compared to larger urban facilities. However, it was surprising that *Supervisor/Manager expectations & actions promoting patient safety* did not enhance effective handover of department accountability. Supervisors/managers may need to model the desired behaviour, and provide recognition or incentives for staff performing effective handover.

Within this current study respondents believed that some staff have a poor understanding of the roles of other health professionals and a lack of acknowledgement of the care that other departments can/could deliver. This is supported by other studies that identified communication of clinical information during handover as a source of staff dissatisfaction, resulting in stress and frustration [2]. Studies indicate that rural health providers are keen to provide safe care and that rural practitioners often have a network of relationships that can and should support effective communication [37, 38]. However, more needs to be done to support the use of systematic approaches to handover of information. Translational research is required to understand barriers and enablers to their use in rural contexts. Ensuring that members of inter-professional teams are aware of each other’s roles, abilities and capacity to assist in providing handover that optimises the effective utilisation of information and hence patient outcomes. There is also evidence to suggest that patient safety can be enhanced through cultural change that encourages and supports patient and family participation in bedside handover. Bedside handover provides an opportunity for patients to understand more about their care, ask questions and to identify and add any missed information, and to be involved in decisions about their care. It has also been shown to support inter-professional as well as intra-professional communication about the patient’s health, care plan and progress [39].

### Limitations

These results must be interpreted in light of the fact that the results are presented as an amalgamation of participants across six LHDs in order to preserve anonymity: We did not analyse results for individual LHDs. It may well be the case that LHDs performed differently on each of the factors. In spite of the large sample size, the number from each LHD is small. The response rates (between 2 and 28.5%) in some LHDs were very low and the results should be interpreted with caution. The sub-scale “Frequency of events reported” of the original HSOPSC was replaced with new items. Therefore, the reliability and validity of this scale needs further assessment.

## Conclusion

This study has highlighted the critical relationship between patient safety and handover in rural health care facilities in Australia. Findings point to the need for approaches to handover that take into consideration the particular challenges associated with rurality and for close examination of approaches and practices that are perceived as working well, for example bedside handover. Further research will need to take into account patient and family experiences in order to understand what effective handover would look like for them. Handover, as an essential element of effective care, is particularly challenging in rural areas. Handover at transitions within and across facilities is an area where future research and strategic improvement is required, along with attention to the place and practice of communication via electronic media. Effective handover can only be enhanced through organizational support and structures, together with individual health professionals taking responsibility to improve communication networks and processes. However, it is important to recognise that the one model may not work in all rural facilities.

## Additional file

Additional file 1: Early and later respondents’ characteristics. Table comparing characteristics of early and late survey respondents. (DOCX 13 kb)

## Abbreviations

AHRQ: Agency for Healthcare Research and Quality
HSOPSC: Hospital Survey on Patient Safety Culture
iSoBAR: Identify, Situation, Observation, Background, Agree to Plan, Readback
LHD: Local Health District
NSW: New South Wales
RTTDC: Rural Trauma Team Development Course
SAC: Severity Assessment Codes
