# Supplementary material for: The impact of patient safety culture on handover in rural health facilities
Source: BMC Health Serv Res. 2018 Nov 26;18:889. doi: 10.1186/s12913-018-3708-3 (PMC6257960; doi:10.1186/s12913-018-3708-3)
Supplement: Supplementary file 1 — Early and later respondents’ characteristics. Table comparing characteristics of early and late survey respondents. (DOCX 13 kb) [file 12913_2018_3708_MOESM1_ESM.docx]

Additional file 1 Early and later respondents’ characteristics

|  | Wave 1 (%)  (*n* = 1007) | Wave 2 (%)  (*n* = 580) | P value |
| --- | --- | --- | --- |
| Age |  |  |  |
| 18-24 | 3.5 | 4.0 |  |
| 25-34 | 11.7 | 12.8 |  |
| 35-44 | 18.6 | 14.8 |  |
| 45-54 | 35.5 | 32.6 |  |
| 55-64 | 29.1 | 33.8 |  |
| 65+ | 1.6 | 2.0 |  |
| Sex |  |  |  |
| Female | 84.9 | 84.7 |  |
| Male | 14.7 | 14.8 |  |
| Other | 0.4 | 0.5 |  |
| Local Health District |  |  |  |
| A | 20.3 | 18.9 |  |
| B | 23.6 | 6.2 | < 0.05 |
| C | 17.9 | 23.8 | 0.005 |
| D | 9.8 | 13.8 | 0.017 |
| E | 4.7 | 4.9 |  |
| F | 23.6 | 32.4 | < 0.001 |
| Type of facility |  |  |  |
| Multi-purpose facility | 12.5 | 10.4 |  |
| Community Health | 25.4 | 21.1 |  |
| District Hospital | 32.6 | 37.5 |  |
| Rural Referral Hospital | 15.7 | 15.8 |  |
| Tertiary Referral Hospital | 3.2 | 3.8 |  |
| Other | 10.5 | 11.5 |  |
| Hours worked per week |  |  |  |
| < 20 hours | 6.2 | 6.4 |  |
| 20-39 hours | 45.1 | 44.1 |  |
| 40+ hours | 30.8 | 18.1 |  |
| Number of years worked in facility |  |  |  |
| < 1 year | 9.0 | 12.5 |  |
| 1-5 years | 31.5 | 32.4 |  |
| 6-10 years | 21.1 | 17.4 |  |
| 11-15 years | 15.4 | 10.3 | 0.018 |
| 16-20 years | 9.7 | 9.1 |  |
| 21+ years | 13.2 | 18.2 | 0.026 |
| Main staff position |  |  |  |
| Medical | 3.7 | 4.2 |  |
| Nursing | 50.6 | 53.0 |  |
| Allied Health | 20.5 | 18.2 |  |
| Management/Administration | 16.7 | 16.9 |  |
| Other | 8.6 | 7.7 |  |
| Primary work area |  |  |  |
| Many different units/no specific units | 19.3 | 17.9 |  |
| Acute inpatient non-surgical | 3.6 | 2.7 |  |
| Acute inpatient surgical | 4.0 | 5.0 |  |
| Sub-acute | 1.9 | 1.6 |  |
| Obstetrics | 4.2 | 5.2 |  |
| Paediatrics | 2.0 | 2.0 |  |
| Emergency | 4.0 | 8.1 | 0.002 |
| Intensive care unit | 2.2 | 1.6 |  |
| Community Health | 19.2 | 12.9 | 0.005 |
| Mental Health | 10.1 | 12.2 |  |
| Rehabilitation | 1.6 | 0.9 |  |
| Pharmacy | 0.6 | 0.5 |  |
| Pathology | 0.6 | 2.9 | 0.001 |
| General Ward | 7.2 | 6.8 |  |
| Radiology | 2.5 | 2.9 |  |
| Recovery | 0.5 | 2.3 | 0.005 |
| Allied Health | 11.4 | 10.9 |  |
| Outpatients | 5.1 | 3.6 |  |
